# Hemodynamics in Cardiac Development

**DOI:** 10.3390/jcdd5040054

**Published:** 2018-11-06

**Authors:** Robert E. Poelmann, Adriana C. Gittenberger-de Groot

**Affiliations:** 1 Department of Animal Sciences and Health, Institute of Biology, Sylvius Laboratory, University of Leiden, Sylviusweg 72, 2333BE Leiden, The Netherlands; 2 Department of Cardiology, Leiden University Medical Center, Albinusdreef 20, 2300RC Leiden, The Netherlands; a.c.gitten@lumc.nl

**Keywords:** cardiogenesis, endocardial cushions, neural crest, hemodynamics, shear stress, semilunar valve, outflow tract septum, Klf2, growth factors, TGF beta

## Abstract

The beating heart is subject to intrinsic mechanical factors, exerted by contraction of the myocardium (stretch and strain) and fluid forces of the enclosed blood (wall shear stress). The earliest contractions of the heart occur already in the 10-somite stage in the tubular as yet unsegmented heart. With development, the looping heart becomes asymmetric providing varying diameters and curvatures resulting in unequal flow profiles. These flow profiles exert various wall shear stresses and as a consequence different expression patterns of shear responsive genes. In this paper we investigate the morphological alterations of the heart after changing the blood flow by ligation of the right vitelline vein in a model chicken embryo and analyze the extended expression in the endocardial cushions of the shear responsive gene Tgfbeta receptor III. A major phenomenon is the diminished endocardial-mesenchymal transition resulting in hypoplastic (even absence of) atrioventricular and outflow tract endocardial cushions, which might be lethal in early phases. The surviving embryos exhibit several cardiac malformations including ventricular septal defects and malformed semilunar valves related to abnormal development of the aortopulmonary septal complex and the enclosed neural crest cells. We discuss the results in the light of the interactions between several shear stress responsive signaling pathways including an extended review of the involved Vegf, Notch, Pdgf, Klf2, eNos, Endothelin and Tgfβ/Bmp/Smad networks.

## 1. Introduction

Heart development requires complex interactions starting with the mesodermal cardiac crescent and resulting in birds and mammals in the fully septated four-chambered beating heart. This complexity relies strongly on the concerted spatio-temporal regulation of many genes. It is evident that a rather limited number of cardiac transcription factors, more specifically Tbx5, Nkx2.5 and the Gata-family, govern major developmental steps, and mutations in these genes have been reported to lead to congenital heart defects (CHD). The pathways reveal highly complex backgrounds including e.g., dose-dependency, histone modifications, copy number variants and post-transcriptional regulation by e.g., microRNAs [1]. The majority of CHD (~80%), however, is considered multifactorial, implying that other, probably environmental, factors are also involved. These include cholesterol metabolism, homocysteine, maternal diabetes, hyperglycemia and hemodynamics. 

Simple changes in the venous blood flow to the model chicken heart [2] result in a variety of ventricular septal defects (VSDs) and pharyngeal arch arterial malformations. Altered gene expression patterns have been found for flow-dependent Et1 (endothelin), eNos and Klf2 [3], bound to the lining layers of the cardiovascular system, i.e., the endocardium and the endothelium.

Obviously, heart development engages not only the shear stress-sensitive endothelial/endocardial inner lining but also myocardial, epicardial, smooth muscle and neuronal cells, and fibroblasts derived from their precursors. These involve development and differentiation of the pharyngeal endoderm, first heart field (FHF), second heart field (SHF) and cardiac neural crest (NC), interacting harmoniously to construct the various components and compartments of the heart. Transmission of hemodynamic cues to the underlying cells (myocardium derived from the SHF, fibroblast and smooth muscle cells derived from the epicardium, [4]) must be an important function of the endothelium and endocardium, and this has been confirmed at least for the Et1 and eNos pathways [5]. The mesenchymal contents of the atrioventricular (AV) and outflow tract (OFT) endocardial cushions have a cushion-specific origin, being from the endocardial lining through endocardium–mesenchymal transition [6], the epicardium [7,8,9] and also from the NC [10,11] and the SHF [12,13].

An important morphogenetic event in heart development is the differentiation of the endocardium into mesenchymal cells, the so-called endocardial–mesenchymal transition [14] (EMT), by which both the AV and the OFT endocardial cushions will be formed. These cushions, located prominently in the bloodstream, function as backflow-preventing structures only transforming into definitive valvular leaflets in later phases. Furthermore, they are instrumental in septation processes in the atrium, ventricle and OFT. As a histological consequence, the underlying cells derived from endocardium, SHF or NC, are in intimate contact with the overlying endocardium (in the case of the heart) and endothelium (in the case of the aortic sac and pharyngeal arch arteries).

Besides being influenced by hemodynamics, genes might also be caught in webs of other interactions exemplified by the Sonic hedgehog (Shh)–Gli pathway, that is shear stress dependent being part of the shear-sensing primary cilium [15], but also involved in cholesterol metabolism [16]. To carry this even further interactions between Shh–Gli with Zic3, Pitx2 and Nodal are important in left–right asymmetry [17] while Nodal–Pitx2 interactions are also involved in asymmetrical development of the pharyngeal arch arteries. This asymmetry changes the blood flow in the aortic sac leading to differential signalling by shear stress responsive genes such as *Pdgf Receptor* as well as *Vegf Receptor-*2 for further downstream arterial remodelling [18].

Essential for unraveling the influence of hemodynamics is that normal flow patterns can be investigated and modeled in vitro and in vivo during development and compared to structural alterations as performed in accessible embryos like chicken [19,20,21,22] and zebrafish [23,24].

In this paper we will provide new information on the effect of ligating a vitelline vein in an experimental chicken embryo on the morphogenesis of the endocardial cushions and the expression of a shear stress responsive gene (*Transforming growth factor Receptor III*). The ensuing behavior of endocardial and NC cells is analyzed, showing malformations in the heart under hemodynamic challenging. 

We will blend the results of shear stress sensitivity on changes in gene expression patterns in a broader sense including a literature review of the involvement of essential shear stress-responsive gene networks in cardiac development, as brought to us by several different approaches. These include: (i) cell culture systems, (ii) in vivo transgenic technology, (iii) the survey of (cardiovascular) patient populations and (iv) direct (surgical) manipulations in chicken embryos.

## 2. Cardiac Anomalies after Vitelline Vein Ligation

### Materials and Methods

Ligation procedures. Fertilized white leghorn eggs (n = 246) were incubated (70 h) reaching Hamburger Hamilton stage (HH) 17. The egg shell was windowed and the vitelline membranes removed above the most proximal part of the right lateral vitelline vein. A small incision was made to expose the vein and a nickel microclip was clamped around it. Cessation and subsequent rerouting of blood flow was confirmed. Eggs were resealed, re-incubated and sacrificed at successive stages. Shams (n = 14) (no ligation) and normal eggs (n = 10) served as controls. Gross morphology and heart morphology and function were evaluated.

Immunohistochemistry. Embryos (n = 169) were fixed (4 °C, overnight) in 98% ethanol containing 2% glacial acetic acid, dehydrated in ethanol, embedded in paraffin and serially sectioned at 5 µm. The sections were mounted, air-dried, rinsed twice (15 min) in phosphate buffered saline (PBS) and once in PBS supplemented with 0.05% Tween-20. Myocardial cells were stained with anti-muscle actin (HHF-35; DAKO, Carpentira, CA, USA), diluted 1:1000, overnight at room temperature. Ten ligations and 6 controls were studied for the expression of Tgfβ Receptor III (TBRIII) used as shear stress marker. Staining was performed with the primary antibody TBRκ, anti Tgfβ Receptor type III (kindly provided by Dr. J.V.Barnett, Nashville, TN, USA), 1:50 diluted in PBS/Tween-20 and 1% ovalbumin. The sections were rinsed twice (15 min) in PBS and once in PBS/Tween-20 (Sigma Aldrich, Darmstadt, Germany) (15 min) and incubated (2 h) with the 2nd antibody, 1:300 diluted rabbit anti-mouse horseradish complex (Dako P0260), rinsed (3 × 10 min) in PBS and exposed (10 min) to 0.04% diaminobenzidine tetrachloride (Merck KGaA, Darmstadt, Germany) in 0.05 M TRIS-Maleic acid (pH 7.6) with 0.07% imidazole and 0.06‰ H_2_O_2_. The reaction was stopped in PBS. The sections were counterstained with Mayer’s hematoxylin (Merck, Darmstadt, Germany) (5 s) and covered in Entellan.

Scanning electron microscopy (SEM). The right lateral vitelline vein of 68 embryos was ligated and investigated in stages before (HH18–24, n = 31) and after ventricular septation (HH34 and 37, n = 37). The hearts were perfusion-fixed with a mixture of 2% paraformaldehyde and 2% glutaraldehyde in 0.1 M sodium cacodylate (pH 7.2, 4 °C) and stored overnight. Preseptated hearts were opened frontally and septated hearts were opened transversally immediately caudal to the atrial appendages. The hearts were rinsed (0.1 M cacodylate buffer, pH 7.2) and postfixed (1% OsO_4_, same buffer, 4 °C), and dehydrated in graded ethanol. Preparations were critically point dried over CO_2_ by conventional methods, mounted on aluminium stubs, sputter-coated (Balzers MED 010) with gold (3 min) and studied with the Philips SEM 525M.

Labeling of Neural Crest (NC) cells. NC cell tracing has been described elsewhere [10]. In brief, we used specific pathogen-free eggs, free of helper virus. The eggs were windowed at HH stage 9–10. The open neural groove was flushed gently with a solution containing the polycation polybrene (80–100 µg/mL, Sigma, St. Louis, MO, USA) and a replication-incompetent retrovirus containing the bacterial *LacZ* reporter gene). The eggs were resealed and reincubated until HH17. Only normal embryos were used further for vitelline vein ligation (see above). The embryos were reincubated until HH34–37. Embryos (n = 13) were fixed in paraformaldehyde 4% and stained overnight with X-gal [10]. Non-ligated retrovirally infected embryos (n = 16) served as controls.

Apoptosis. To investigate the presence and distribution pattern of apoptotic (NC) cells we subjected retrovirally infected embryos to the TUNEL approach (Tdt-mediated dUTP nick end labeling) using a commercially available kit (Boehringer, Mannheim, Germany) to detect fragmented DNA [10]. After counterstaining, sections were dehydrated and mounted in Entellan.

Survival rates. The survival rate after venous ligation was almost 79% (compared to an estimated survival rate of 90–95% of established fertilized, unopened eggs). We observed that stages HH22–24 were critical in relation to survival.

Fertilized eggs are not considered ‘experimental animals’ under the Dutch law, requiring no specific permits for handling.

## 3. Results

### 3.1. Impaired Development in Preseptation Stages and Tgfβ Receptor III (TBRIII) Expression

**Figure 1:**
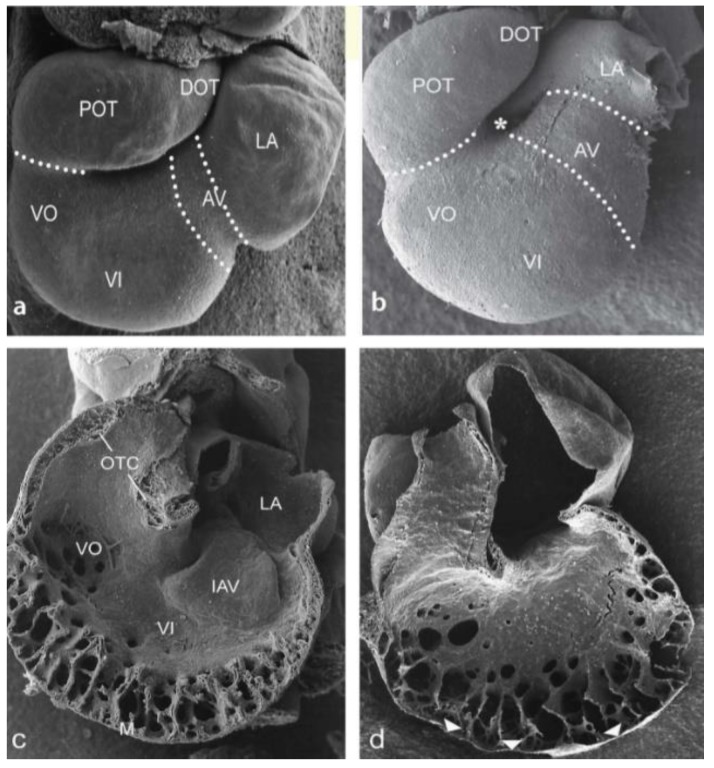
Cardiac looping in normal and ligated embryos HH20. Scanning electron micrograph (SEM) of ventral views. (**a**) Normal embryo with cardiac segments indicated. (**b**) Ligated embryo. The retarded looping resembles that of a HH17 embryo with an open inner curvature (*). The AV canal is relatively long. (**c**,**d**) Interior view of dorsal heart halves. (**c**) The inferior AV cushion and the OFT cushions are well developed, ventricular trabeculations have formed. (**d**) AV and OFT cushions are non-existent, spongy trabeculations and the compact myocardium is thin (arrowheads). AV: Atrioventricular groove, DOT: distal OFT, IAV inferior AV cushion, LA: left part of atrium, M: compact myocardium, OTC: OFT cushions, POT: proximal OFT, VI ventricular inlet, VO: ventricular outlet, * inner curvature, arrowheads: thin compact myocardium.

Normal hearts showed a relatively short AV junction (Figure 1a) compared to ligated embryos (Figure 1b). For an evaluation of the observed cardiac abnormalities in preseptation stages see Table 1. After ligation the inner curvature was wider creating a larger distance between OFT and AV area (compare Figure 1a,b). In normal and sham-operated embryos, numerous mesenchymal cushion cells resulting from EMT were seen and the endocardium covering the cushions was squamous. In ligated embryos, the cushions lacked many cells, where they accumulated directly under the cuboidal lining. Hypoplastic AV cushions were the most common malformations (38%) in ligated embryos (N = 63) in stages HH18–24 (compare Figure 1c,d). The superior cushion was affected more frequently and the effects were more severe than in the inferior one. Hypoplastic OFT cushions were observed in 17% of the ligated embryos (Figure 1d).

**Figure 2:**
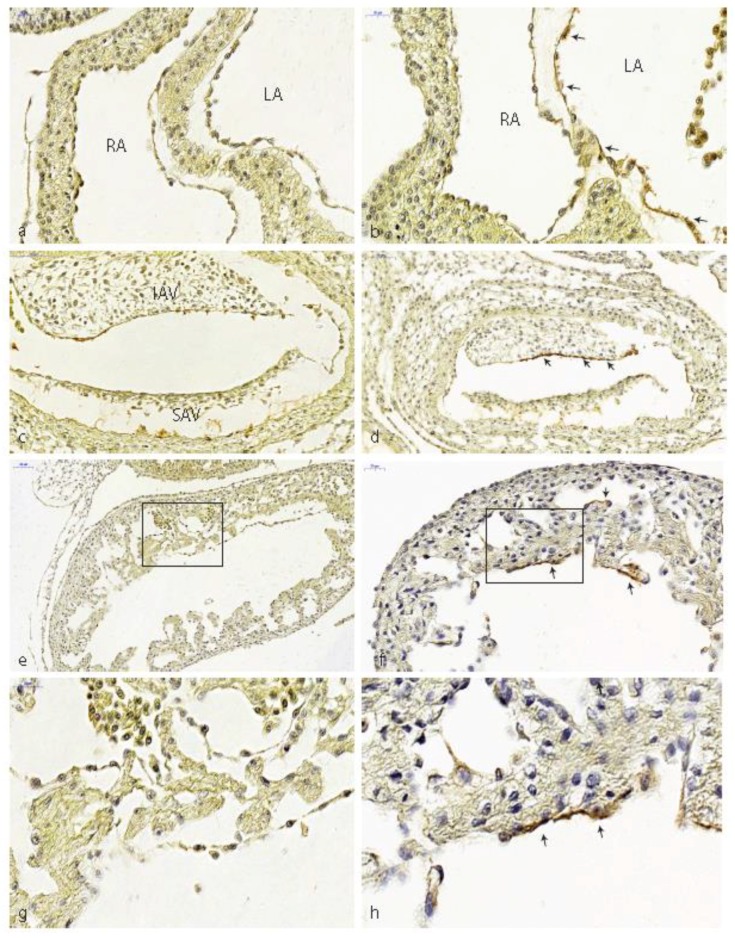
Expression of TGFβ type III receptor. (**a**) Normal left and right atrium of HH20. No TBRIII expression in atrial endocardium. (**b**) Ligated embryo with ectopic TBRIII expression along the atrial septum and atrial floor (arrows). (**c**) AV cushions of normal HH22 embryo, TBRIII expression is downregulated. (**d**) Ligated embryo with prolonged endocardial TBRIII expression (arrows). (**e**) Normal HH20 embryo, ventricular trabeculations lack TBRIII. (**f**) Ectopic TBRIII expression of the endocardium lining ventricular trabeculations after ligation (arrows). (**g**) Higher magnification of the boxed area of (**e**). (**h**) Higher magnification of the boxed area of (**f**). IAV: inferior AV cushion, LA: left atrium, RA: right atrium, SA: superior AV cushion.

In normal HH18 embryos, TBRIII expression was observed in the endothelium of the dorsal aorta, cardinal vein, pharyngeal arch arteries, the endocardium covering the AV and OFT cushions, the epicardium and mesonephros (see for a further description [25]). From HH20 onwards the expression in cushion endocardium was absent, while mesenchymal cells continued to express TBRIII although at a lower level than that seen in ventricular endocardial cells. Venous ligation resulted in HH20–24 in expansion of TBRIII expression toward additional endocardial cells including those lining the atrial septum (compare Figure 2a,b), the floor of the left atrium and the ventricular trabeculae (Figure 2e,f and their higher magnifications (boxed areas) in Figure 2g,h). Furthermore, there was sustained expression in the AV cushion endocardium, especially in the inferior AV cushion beyond HH20 (compare Figure 2c,d).

### 3.2. Peri- and Post-Septation Stages

**Figure 3:**
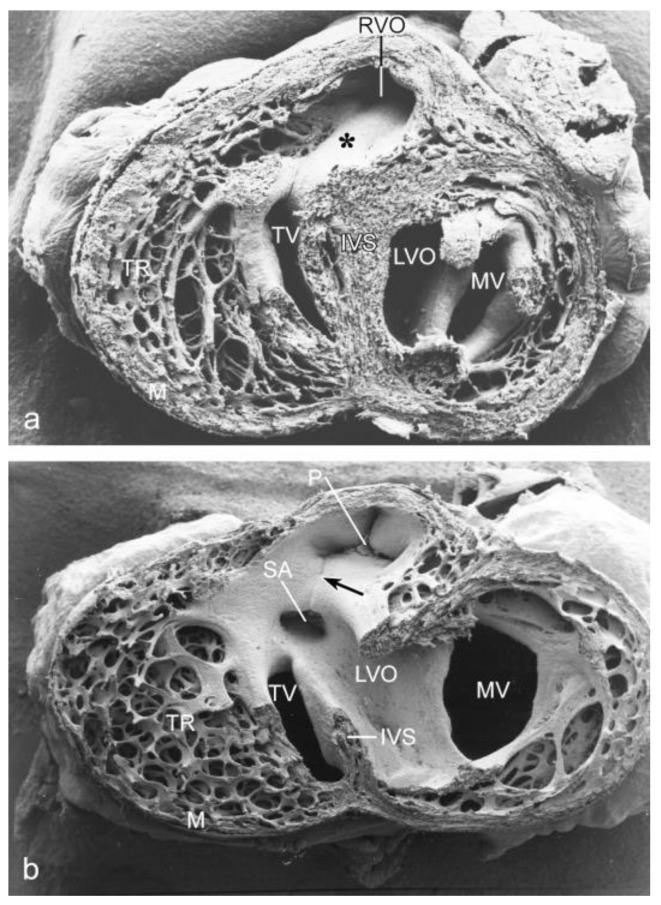
SEM of HH34 postseptation embryos viewed from apex to base. (**a**) Normal heart. The subpulmonary infundibulum (*) is continuous with the interventricular septum (IVS). (**b**) Ligated embryo with a subaortic ventricular septal defect (VSD). The line of the fused OFT cushions is indicated (arrow). Both arterial orifices (SA and P) are situated above the right ventricle (double outlet right ventricle). The aorta is stenotic (SA) and the mitral valve leaflets (MV) are abnormal. The myocardium is severely affected, as seen by a thin IVS and compact layer (M) and spongy trabeculations (TR). IVS: interventricular septum, LVO: left ventricular OFT, M: compact myocardium, MV: mitral valve, P: pulmonary orifice, RVO: right ventricular OFT, SA: stenotic aorta, TR: trabeculations, TV: tricuspid valve.

**Figure 4:**
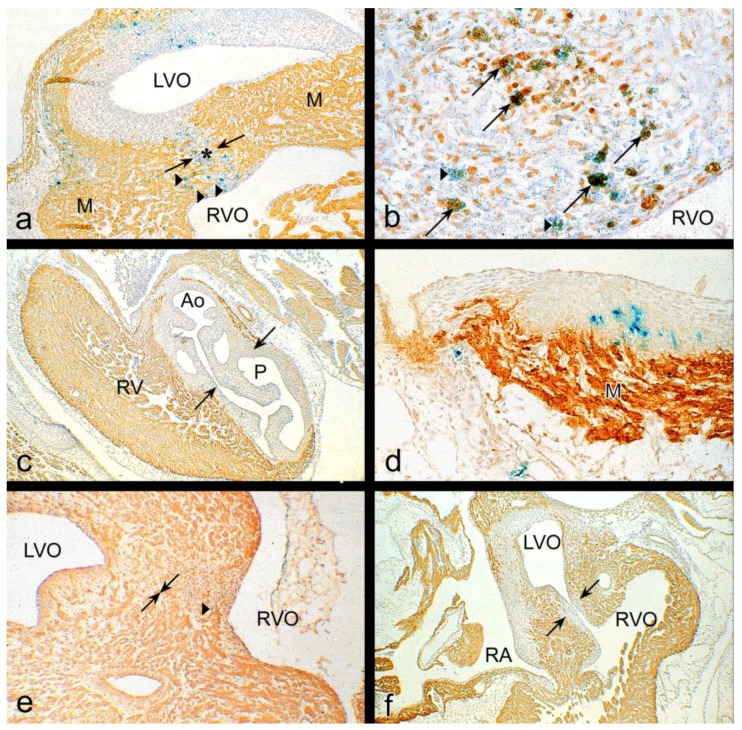
LacZ tracing and apoptosis of neural crest (NC) cells immunostained for actin. (**a**) Proximal OFT region of a retrovirus infected, non-ligated HH31 embryo. Myocardialization of the OFT is nearly complete (*) as the opposing parts of the myocardium (M) almost touch (arrows). NC cells (blue) are present in the mesenchyme and myocardium (arrowheads). (**b**) Adjacent section with magnified AP septal complex, subjected to Tdt-mediated dUTP nick end labeling (TUNEL) for apoptotic cells (brown). Most of the apoptotic (brown) cells are also blue, indicating NC cells. After apoptosis, X-gal granules give way the position of the original NC cell (arrows). (**c**) HH37 ligated embryo with a subarterial VSD, showing confluence of the semilunar valve leaflets without myocardialization (arrows far apart). (**d**) Retrovirus infected, ligated HH37 embryo. Numerous blue NC cells in the ventral prong of the AP-septal complex. Adjacent TUNEL stained sections (not shown) presented no apoptotic cells. (**e**) Retrovirus infected, non-ligated HH37 embryo with full myocardialization (arrows meet each other) only a single blue NC cell (arrowhead) has been registered. (**f**) Ligated HH37 embryo showing a subaortic VSD with substantial myocardialization of the AP septal complex (compare with (**c**)). Ao: aorta, LVO: left ventricular OFT, M myocardium, P: pulmonary trunk, RA: right atrium, RV: right ventricle, RVO: right ventricular OFT.

In HH34 the muscular subpulmonary infundibular wall and the small muscular OFT septum are continuous with the interventricular septum by which the short left and long right ventricular OFT are fully separated (Figure 3a). After ligation, a VSD (Figure 3b) is found in 66% of the embryos. Both arterial orifices may be above the right ventricle (double outlet right ventricle). The aorta is stenotic and the mitral valve leaflets are abnormal. The myocardium is severely affected, as seen by a thin interventricular septum, a thin compact layer and spongy trabeculations (compare Figure 3a,b). After serial sectioning and LacZ tracing, the histology makes this even more clear (Figure 4). In normal embryos the OFT septal complex has contributed to a separation of the arterial trunks, the arterial orifices and the intramyocardial right and left ventricular OFT. In this, the NC cells were found in the condensed mesenchyme and the distal endocardial cushions at the future orifice and semilunar valve level as well in the proximal cushions in the myocardial part of the OFT. These NC cells can be traced using the retroviral-LacZ method (Figure 4a,e). In control embryos many or perhaps all of the NC cells go into apoptosis (Figure 4b). After hemodynamic challenging by the venous clip ventricular septation in about two-thirds of the investigated embryos in HH27–45 was not complete. The resulting VSDs were in the majority of cases of the subarterial type with a typical pathomorphology in which the most distal border of the VSD was at semilunar valve level (Figure 4c). The arterial trunks were separated, but at the level of the arterial orifices and the distal cushions the condensed mesenchyme was ventrally displaced and the proximal OFT cushions were incompletely fused explaining the VSD. At these levels quite a number of NC cells could still be detected (Figure 4d), adjacent TUNEL stained sections presented no apoptosis. In 41% of these surviving ligated embryos the aorta was dextroposed resulting in a double outlet right ventricle. This is most probably linked to the incomplete looping observed in preseptation stages (Figure 1b). We observed a continuous spectrum of deficient OFT septum myocardialization with in the most extreme cases solely a small mesenchymal septum at orifice level, resulting in an annotation for part of these subarterial VSDs as doubly committed. Semilunar valve malformations were frequently encountered (25/70) in combination with a VSD (Figure 4c,f).

## 4. Discussion

In ligated embryos we observed hypoplastic and even absent AV cushions in 38% of the survivors of all preseptation stages (HH18–24). Normally, the cushions fulfill a valve function preventing backflow. We suppose that ligated embryos with severe hypoplastic AV cushions were not able to maintain appropriate cardiac output and died before or near HH22–24. The severe cases are incompatible with development by HH24, which would explain the rare presence of AV anomalies in post-septation stages.

Proper AV and OFT endocardial cushion formation starts with EMT of the overlying endocardium in which many gene pathways are involved [14]. Tgfß signaling [25] is a shear stress-dependent mechanism initiating and supporting this transformation [26]. Several cell types showing an intricate interaction are involved in the remodeling of this region. The OFT endothelial/endocardial cells regulate NC and SHF morphogenesis via an in mouse and chicken conserved signaling circuit involving TGFβ, regulating extracellular matrix remodeling [27]. Furthermore, morphogenesis comprises FGF and TGFβ cross-talk between SHF, NC and myocardium with as pivot the extracellular matrix [28]. The involvement of TBR III suggests a role for Tgfß2, since this ligand requires TBRIII for high affinity binding [29]. A functional role for Tgfß2 is further supported by valve defects and a non-myocardialized OFT septum in a Tgfß2 null mouse [30,31]. Since venous ligation results in alteration in endocardial morphogenesis and valve formation, the finding of persisting TBR III expression supports its role in diminished cushion formation. Normally TBR III is downregulated after the cushions have been seeded with mesenchymal cells [32]. Here, this downregulation does not take place together with diminished seeding of the cushions. Hypoplastic AV cushions, compact myocardial wall thinning and spongy trabeculation correlated well with previous hemodynamic measurements [33]. Hearts probably reacted to decreased function by ventricular dilation which in turn impaired cardiac looping, a key mechanism in the formation of VSDs [1]

*Peri-/post-septation stages*. During development the types of the observed malformations changed. All ligated embryos were abnormal immediately after ligation, while from HH24 onwards, also apparently normal embryos were encountered. Moreover, a number of VSDs closed spontaneously, which can also be observed in human preclinical care.

Here, we have to take into consideration the dynamics of OFT remodeling. This results in 1. considerable shortening and myocardialization of the proximal level and separation of the aortic and pulmonary flow channels; 2. formation of the aortic and pulmonary semilunar valves; and 3. differentiation of the aortic and pulmonary walls [34]. Usually, the structures involved in the separation have been referred to as the NC-dependent aortopulmonary (AP) septum. As other mesenchymal cells (most likely second heart field derived) take part in this structure, it has also been referred to as the AP septal complex [11]. However, due to the extensive remodeling, we have adopted as a proper definition the term OFT septal complex, embracing the changing anatomy over the complete length of the OFT from early development to its final state.

During normal development after arrival in the OFT septal complex, all NC cells become apoptotic [10,35], and subsequently the mesenchyme is replaced by the myocardium [36]. Abnormalities in the OFT septal complex after ligation were recognizable as a ventral displacement of the central mass of the condensed mesenchyme [2], as supported by LacZ tracing of NC cells (this study). Furthermore, in ligated embryos we could hardly observe apoptosis explaining the continued presence of NC cells. These phenomena lead to an exclusively mesenchymal OFT septum suggesting that apoptosis of NC cells is important for myocardialization [37]. The programmed death of NC cells in a limited time frame [35,37] still presents an enigma but we favor the following chain of events. Normally, NC cells migrate into the assigned cardiac regions and become apoptotic, thereby changing the (extracellular) microenvironment. In vitro NC cells produce a latent form of Tgfß [38], also abundantly present in the matrix of embryonic hearts [39] that could be activated by NC proteolysis, and is diminished after venous ligation because of a diminished apoptosis. In this experimental setting, proper endocardial signaling, effected by hypoplastic OFT cushions, is defective. This may lead to altered interactions with NC and SHF cells, an essential condition of arterial pole morphogenesis [27,28], with surviving NC cells as one of the outcomes. Tgfß signaling is, furthermore, important in OFT myocardialization as migration [40] and differentiation [41] of cardiomyocytes is controlled by Tgfß. In Tgfß-2 null mice the OFT septum is not myocardialized [30,31]. It is evident that further research on the role of TGFß signaling in the arterial pole is wanted.

The semilunar valve malformations after venous ligation are difficult to compare with other models as those have rarely been described. The most severe semilunar valve malformations in our model are combined with allegedly NC-related subarterial VSDs, confirming the involvement of NC cells in semilunar valve development [42].

### 4.1. Hemodynamic Load

Hemodynamic patterning has been approached by computational methods in both chicken and mouse [22,43,44] demonstrating the influence of mechanical regulation of the various cardiac compartments and valves. In addition optical methods have been applied to demonstrate flow patterns in chicken and zebrafish [21,24]. Therefore, it is likely that interfering with hemodynamic load, by either decreasing or increasing blood pressure and flow velocities, usually result in cardiac malformations that are quite comparable albeit with varying incidence and severity [45,46,47]. Ligation of a vitelline vein or the left atrium (decrease) or OFT banding (increase) all interfered with looping, ventricular septation and valve formation, but also aortic arch artery morphogenesis [48]. The myocardial architecture changes considerably during development and will become abnormal after hemodynamic intervention [49,50]. At a cellular level, increased hemodynamic load by OFT banding enhanced EMT. In the OFT cushions, the cell density increased and extracellular matrix constituents were severely altered [51]. A different approach has been used by increasing regurgitant flow through optic pacing at HH13 to 180 beats/minute [52]. A significant number of embryos presented severe endocardial cushion defects, often being lethal, demonstrating that hypoplastic cushions are incompatible with survival.

### 4.2. Ciliary Mechanosensing

Changes in the cellular environment are perceived by many cell types through e.g., integrins [53] and receptive organelles such as non-motile monocilia or primary cilia [54], important for various aspects of shear stress-dependent signaling during embryonic development [55]. Monocilia are not only involved in shear stress mediation but in many other processes, revealing diverse regulatory inputs [56]. Mechanical activation of the cilium evokes trafficking along the ciliary membrane and the enclosed microtubular system, resulting in intracellular signaling [57]. Several ciliary genes themselves are shear stress responsive, such as *Aurora*, Intraflagellar transporters and the homodimeric platelet-derived growth factor receptor (*PdgfRαα*) [58]. In the cardiovascular system endothelial and endocardial cells present monocilia [59,60], particularly in areas of low shear stress [61,62].

Monocilia are relatively abundant in the trabecular sinuses and in those curvatures where the flow and the ensuing shear stress is low, such as the outer curvature of the aortic arch [63,64] and even in the adult mouse where monocilia are also abundant in the aortic valve sinuses and more downstream near branching points of e.g., the carotid arteries. In Apo3-/- mice the monocilia are found on the shoulders of atherosclerotic lesions in areas of turbulent flow, suggesting a relation between hemodynamics, monocilia and lesion formation [63]. Experiments using cultured endothelial and endocardial cells in flow chambers demonstrate that the (dis)appearance of monocilia is highly dynamic and subject to many factors [26].

### 4.3. Gene Expression Patterns in Mechanosensing

It is obvious that an embryo does not exist as a separate entity, but is connected to its yolk sac (and in mammals also the placenta), the blood volume of these embryonic/fetal organs differs in development but is certainly not negligible. Therefore, it is no surprise that manipulating blood flow coming from the yolk sac will have major implications on cardiovascular development and may even be lethal [65]. Ligation of yolk sac vessels results in altered hemodynamics [20,33], succeeded by upregulation of Klf2 and Nos3, but by downregulation of Et-1 [3]. Increasing the hemodynamic load of the developing heart by OFT banding [51] resulted in increased cellularity of the OFT cushions and changes in the extracellular matrix combined with altered expression of factors known to be markers of EMT. Among these were Notch, Tgfβ, VegfR and Gata4 [51], all involved not only in matrix metabolism but also in many other aspects of cardiovascular development. In a meta-study, flow-related signalling was confirmed for ~1650 shear stress responsive genes expressed in human umbilical vein endothelial cells (HUVECs) cultured in vitro [58] regulating genes in 24 signalling pathways. In this paper, we concentrate on a subset of factors and signalling pathways that have also been identified in vivo during cardiac development. We will focus on Vegf, Notch, Pdgf, Klf2, Nos3, Endothelin, and Tgfβ/Bmp/Smad.

#### 4.3.1. Vascular Endothelial Growth Factor (Vegf) Signaling 

The Vegf/Vegf Receptor family contains several ligands (Vegf-A, B, C, D and PlGF), binding to Vegf Receptor1 (also named Flt1), R2 (also named Flk1/Kdr), R3, and cofactors as Neuropilin-1 and -2 [66]. Both Vegf-A and Vegf-B are shear responsive in HUVEC [56]. VegfR1 and 2 are expressed on endothelial cells and bind Vegf-A. All Vegf isoforms bind VegfR2, although the presence of a co-factor seems to be involved in regulating specific effects (reviewed in [67]). Several additional pathways are involved such as hypoxia signaling [68] and homocysteine metabolism [69] to keep the phenotype in balance. Vegf polymorphisms and redundant pathways are involved, as shown by the various possible outcomes. These include Tetralogy of Fallot, valvular and septal defects and left ventricular outflow obstruction. It is interesting to note that all cardiac malformations reported in VEGF polymorphisms can be traced back to developmental disorders in endocardium, epicardium or NC cells and their EMT [67,68]. Furthermore, one of the main downstream networks of Vegf/VegfR2 is the Notch-signaling pathway [70].

#### 4.3.2. Notch Signaling

Several members of the endothelial [58,71,72] and endocardial [73] Notch-signaling pathway are shear stress-sensitive including Dll4 (involved in trabeculation and coronary vessel formation), Jag1 and Jag2 (involved in chamber maturation and compaction) and downstream genes such as Hes and Hairy [74]. Perturbation of the signaling balance severely interrupts cardiac chamber formation. Dll4 mutant embryos showed more than 2000 affected genes [73]. Notch signaling in the SHF mediates interactions with the homing NC cells involved in proper OFT development [75]. 

In a human subpopulation, mutations in Jagged1, also involved in Alagille syndrome, result in cardiac defects, including bicuspid aortic valve and Tetralogy of Fallot with dysmorphic pulmonary valve, overriding aorta, VSDs and right ventricular hypertrophy [76]. In animal models, the outcome is complicated due to redundancies in the pathway, while penetrance of the phenotype highly depends on the genetic background [77].

#### 4.3.3. Platelet-Derived Growth Factor (Pdgf) Signaling

Pdgf isoforms consist of homodimers and heterodimers of four chains (A–D). They bind to the Pdgf receptor α and β subunits with varying affinity. Various members of the Pdgf pathway are shear stress responsive, including PdgfRαα, that in fibroblasts is even localized to the primary cilium itself [78]. Dimer signaling often involves intermediate transducers such as (shear stress responsive) Ras and PI3K. Shear stress applied to bovine endothelial cells produces enhanced PdgfRα activation giving a chemotactic response to smooth muscle cells [79]. Dysregulation of the human *PdgfA* gene is associated with total anomalous pulmonary venous return providing evidence that this gene is involved in proper formation of the cardiac inflow tract. This is confirmed in mouse and chicken embryos studying the PdgfRα and its ligand PdgfA [80,81]. PdgfA, -C and its receptor α are involved in remodeling of the compact and trabeculated myocardium as well as development of the AV valves through epicardium-myocardial interaction [81].

#### 4.3.4. Krüppel-Like Factor-2

In adult vessels the mechanical force of shear stress is a strong inducer of Klf2 [58,82,83]. In HUVEC, Klf2 regulates the transcription of many downstream factors in e.g., the Tgfβ signaling pathway [84] and also aquaporin-1, a nitric oxide transporter [85]. Klf2 is expressed in the endocardium of mouse and chicken and heavily involved in normal cardiogenesis [86]. It is engaged in regulating endocardial cell morphology during chamber ballooning. Cell-specific conditional Klf2 knock out mice demonstrated endothelial loss of Klf2, resulting in lethal embryonic heart failure [87]. Klf2 ablation results in reduced Sox9, UDP-glucose dehydrogenase (Ugdh), Gata4 and Tbx5 mRNA in the AV canal [86]. In the chicken embryonic heart, its expression has been shown particularly in areas of high shear forces, which is the inner curvature of the heart, and at narrow regions such as the AV canal and the OFT [5]. Increasing the hemodynamic load by OFT banding resulted in upregulation of Klf2 with a concomitant changed extracellular matrix protein profile and, in particular, a dysmorphic mitral valve [88]. It has to be kept in mind that high shear areas are nearly devoid of primary cilia as these are abundant specifically in low shear areas [63]. Endocardial differentiation defined by expression of Klf2 and Notch1 is dependent on blood flow within the ventricle and the AV canal.

#### 4.3.5. Endothelin Signaling

Endothelin is a small peptide derived from prepro-Et-1 mRNA that becomes translated into a 203-amino acid precursor, converted by endopeptidases into big-Et-1 which becomes cleaved by endothelin-converting enzyme (ECE) into the functional endothelin. This exerts its activity through two main classes of receptors, Et-A and Et-B, present on e.g., smooth muscle and endothelial cells. ECE and Et-B are both shear stress-responsive [58,89] and expression is down-regulated upon applying shear forces [90]. 

Endothelin is an endogenous vasoconstrictor but has also vasodilator properties mediated by nitric oxide and prostacyclin release through activation of the endothelial Et-B receptor. Furthermore, endothelin is a growth factor involved in the proliferation of fibroblasts and smooth muscle cells through the Et-A receptor and the proliferation of endothelial cells through the Et-B receptor (reviewed by [5]). Et-1 mRNA and protein production are regulated by wall shear stress, although the mechanisms are controversial [91]. In early chicken embryos, Et-1 is expressed in the lining of the endocardial cushions, where during development it becomes complementary to that of Nos3 and Klf2 [92]. Et-1 knock out mice display similar cardiovascular defects as seen in chicken embryos after ligation of the vitelline vein [65,93]. Increasing the hemodynamic load by OFT banding in chicken embryos resulted in decreased mRNA expression and dysregulation of extracellular matrix proteins and abnormal AV valve development, particularly affecting the mitral valve [88]. Involvement of Et-1 in the induced abnormalities found after venous ligation is proven by systemic application of endothelin or antagonists of Et-A and Et-B showing also direct hemodynamic changes [92].

#### 4.3.6. Nitric Oxide (NO) Signaling

Nos3, also called endothelial nitric oxide synthase (eNos), is the major isoform in the vascular system, and also expressed in cardiomyocytes, the functional counterpart of Et-1. Both eNos and Nostrin (nitric oxide trafficker) are shear stress-responsive [58]. In vitro laminar shear stress promotes NO-formation and increases the expression of Nos3 [94,95]. The synthase is mainly localized in plasmalemmal vesicles, caveolae, where it is involved in the production and release of the bioactive NO. Nos3 catalyzes the conversion of L-arginine and oxygen to L-citrullin and NO. Expression depends also on e.g., hypoxia, whereas the balance between NO and Et-1 is physiologically important for maintaining vascular homeostasis. In vivo studies showed that Nos3 expression overlaps with the high shear marker Klf2 [3]. In Nos3-deficient mice, smooth muscle cell proliferation in a carotid artery ligation model is suppressed [96] and they display bicuspid aortic valve, heart failure, VSD and ASD [97]. Furthermore, these mice show hypoplastic coronary arteries already early in development (where it is probably related to the rise in blood pressure and flow at the onset of arterial irrigation), followed by postnatal myocardial infarction. The underlying mechanism is complex as lack of Nos3 results in down-regulation of Gata4, Wilms tumour-1, Vegf, Fgf and erythropoietin and, furthermore, in inhibited migration of epicardial cells [98]. The epicardium is important for coronary formation and differentiation [99]. In chicken embryos its expression increases after ligation of a vitelline vein, just like Klf2 [3,5].

#### 4.3.7. Tgfβ/Bmp Signaling

Members of the Tgfβ superfamily include activins and inhibins, Bmps (bone morphogenetic proteins) and others. Shear responsiveness is confirmed for but not limited to Tgfα, Tgfβ-1, TgfβR-1, latent Tgf binding proteins, Bmp1, 2 and 4 and the Bmp endothelial regulator [58]. Tgfβ1, 2, and 3 exhibit a myriad of regulatory, proliferative, and inductive functions in sometimes distinct but also overlapping spatial and temporal patterns in development as well as in the adult. The Tgfβs are secreted as latent complexes and become activated by e.g., metalloproteinases, and have an interaction with integrins, reactive oxygen species, retinoids and others. Three classes of receptors are distinguished, Tgfβ-RI (Alk 1–7), -RII (including TgfβR2, BmpR2 and activin receptors), and -RIII (including betaglycan and endoglin) with varying degrees of affinity to the ligands [100,101,102]. The TGFβ/BMP downstream signaling pathway involves activated Smad proteins [103].

In vitro applied shear stress resulted in alignment of endothelial cells, diminished apoptosis and proliferation, and increase in Tgfβ3, Klf2, phosphorylation of Nos3 and NO-release. EMT of the endocardium is coordinated by Bmp2 through the activation of genes that regulate intercellular communication, cell adhesion and extracellular matrix deposition [104]). In the OFT, not only endocardial cells migrate into the cushion, but also NC cells expressing TgfßR2 [105], that modulate OFT septation and cushion remodeling. Knockdown of *Tgfß2* results in a variety of cardiac malformations [30,31] while knock down of *Tgfβ3* prevented the induction of Klf2 [106]. In embryonic endothelial cells shear stress activates Tgfβ/Alk5 signaling, while induction of Klf2 is Alk5 dependent [64].

Knock out mice present many phenotypes in major organs including the cardiovascular system (see for an overview of phenotypes [103]) often involving the cardiac jelly and the cardiac cushions.

#### 4.3.8. Interactions between Flow-Responsive Genes

**Figure 5:**
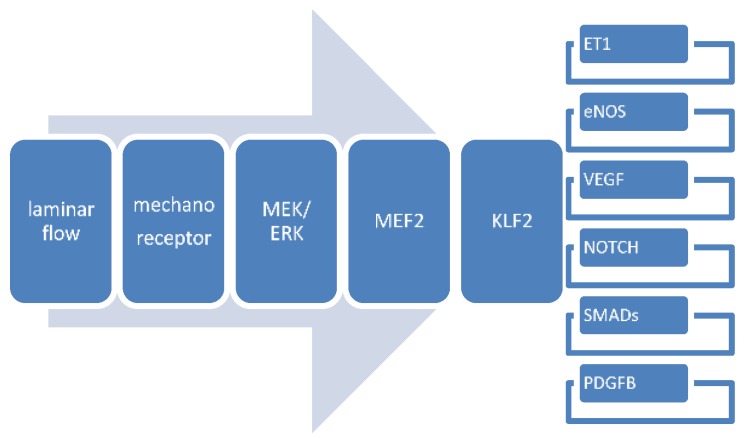
Alteration in laminar flow invokes changes in mechanoreceptors such as monocilia and integrins, followed by activation of e.g., the Mek/Erk-Mef2 cascade. Mef2 activates nuclear expression of *Klf2* thereby influencing *Et-1, eNos*, *Vegf*, *Smads* (particularly *P-Smad2*, and *Smad4/7*) and probably *Pdgf-B*. Upregulation of *Klf2* by changes in laminar flow (but also by Angiopoietin-activation of the Tie2 receptor and by statins) involves down-regulation of most of the latter genes. It is interesting to note that oxidative stress and cytokine but also oscillatory flow not sensed by monocilia downregulate *Klf2* expression. Data mainly based on [3,5,63,105].

The above featured signaling pathways after flow bear many more influences from other factors such as hypoxia (Vegf and eNos), and are part of myriad cascades that are not solely flow-responsive (Vegf and Notch, Tgfß signaling). Furthermore, interactions might be reciprocal as for instance in Pdgf-B and Klf2 in embryonic aortic smooth muscle maturation [107]. The key factor in flow-responsiveness in the adult vessel wall seems to be Klf2 governing a gene transcription profile of >1000 genes [108] involved in cell migration, vasomotor function, hemostasis, inflammation and morphology changes and we propose that Klf2 likewise plays an important role in embryonic development of the cardiovascular system. An all-inclusive scheme showing the (potential) ramifications is hard to conceive; therefore, we provide a simplified version of the main flow-dependent interactions in cardiovascular development (Figure 5).

### 4.4. Consequences for Human Embryonic Development

It is evident that shear stress-responsive genes share pathways in the genesis of malformation complexes. We have shown that endocardial and NC cells take part in the chicken venous ligation phenotype. The avian vitelline circulation used here serves as a model for the mammalian/human umbilico-placental circulation. Whether alteration in placental blood flow in mammals will lead to a similar spectrum of anomalies remains to be determined with advanced ultrasound and Doppler techniques. Retrospective research showed a correlation of human placental anomalies with intrauterine growth retardation [109]. Human cardiovascular malformations are related to low birth weight, body length and head circumference. Furthermore, abnormal circulation may result in growth retardation [110]. It was demonstrated that fetuses with intrauterine growth retardation displayed increased umbilical, placental and utero-placental resistance, decreased end-diastolic flow velocities in the descending aorta and umbilical artery, and decreased peak systolic flow velocities at cardiac level [111]. During prenatal diagnosis using Echo-Doppler techniques it has been shown that malformations specifically of the OFT and aortic arches can aggravate due to diminished blood flow through stenotic regions. Prenatal surgical interventions to relieve the stenosis results in restoration and limitation of the cardiac malformation [111]. Thus evidence is present for a role of hemodynamic factors in the human fetus in growth retardation as well as the emergence of congenital (cardiac) malformations.

## Figures and Tables

**Table 1:** Cardiovascular abnormalities after ligation. The number and percentage of malformations are indicated for each stage. Different malformations were sometimes observed in the same embryo, therefore, the sum of a column can exceed 100%.

Hamburger Hamilton (HH) Stages	8–20		22–23		24		Total 18–24	
Number of Embryos	n = 19	%	n = 16	%	n = 28	%	n = 61	%
Normal	0	0	0	0	77	25	7	11
Disturbed looping	6	32	5	31	9	32	20	32
Hypoplastic atrioventricular (AV) cushions	7	37	8	50	9	32	24	38
Hypoplastic outflow tract (OFT) cushions	3	16	2	13	6	21	11	17
Myocardium left atrium	8	42	4	25	0	0	16	25
Myocardium ventricle	3	16	3	19	6	21	12	19
Pharyngeal arch arteries	1	5	1	6	7	25	9	14
